# Development and validation of the performance evaluation anxiety task: a computerized task to identify functionally significant test anxiety

**DOI:** 10.3389/fpsyg.2026.1819517

**Published:** 2026-07-29

**Authors:** Kenneth A. Winter, Timothy M. Dasinger, Erick A. Bourassa-Price

**Affiliations:** 1 Department of Biology, Mississippi College, Clinton, MS, United States; 2 Department of Advanced Biomedical Education, School of Medicine, University of Mississippi Medical Center, Jackson, MS, United States

**Keywords:** attentional control theory, EEG, performance anxiety, salivary cortisol, test anxiety, validation study

## Abstract

All currently available instruments for measuring test anxiety are subjective surveys that do not assess functional impairment. Using the attentional control theory of anxiety as its theoretical basis, the Performance Evaluation Anxiety Task (PEAT) was designed as a computerized task to objectively quantify performance disruption induced by negative performance evaluation. Normative scores were determined from a pool of 573 participants. The task was validated using four complementary approaches. Salivary cortisol increased significantly more in individuals classified as highly test anxious (HTA) than low test anxious (LTA), demonstrating physiological sensitivity to evaluation (*n* = 207). In a dot-probe task with electroencephalography (*n* = 55), HTA demonstrated altered attentional engagement and disengagement, along with modulation of neural markers of cognitive control (frontal midline theta, theta/beta ratio, posterior theta and alpha power) specifically in response to test-related threat, but not generally threatening items. In a go/no-go task (*n* = 39), HTA exhibited negative performance feedback-induced increases in commission errors and reductions in post-error slowing without differences in overall accuracy, indicating evaluation-specific executive control disruption rather than global performance deficits. Finally, PEAT classification was unrelated to ADHD diagnosis or medication use (*n* = 75), supporting discriminant validity. Taken together, these findings provide convergent behavioral, physiological, and neural evidence of the construct and predictive validity of the PEAT as an objective measure of functionally significant test anxiety.

## 1 Introduction

Test anxiety (TA) is reported to be a common condition (15–50% of the student population) (Cizek and Burg, 2006; Huberty, 2009; Segool et al., 2013; Hanfesa et al., 2020) and in the current test-focused academic climate, it is estimated that the proportion of students with TA will continue to increase (Hembree, 1988; Wren and Benson, 2004). Despite how common TA is reported to be, it is not recognized in the current edition of the Diagnostic and Statistical Manual of Mental Disorders (DSM-5) (APA, 2013). Although TA was considered for inclusion in the DSM, it was ultimately rejected because it had no standard definition or cutoff values on available assessments, there was uncertainty whether test anxiety could be differentiated from other anxiety disorders, and there was no evidence of physiological responses (such as changes in heart rate or blood pressure) in highly test anxious (HTA) relative to low test anxious (LTA) students before and during a test (LeBeau et al., 2010).

There are a number of assessments available that purport to measure TA. The most commonly cited test anxiety assessments are the Test Anxiety/Attitude Inventory (TAI), Westside Test Anxiety Survey (WTAS), Children’s Test Anxiety Scale (CTAS), the Test Anxiety Scale for Children (TASC), and the test anxiety screening tool developed by Nist & Diehl (N&D). Each of these instruments are similar in that they attempt to assess TA using a subjective survey; however, they differ in their theoretical framework, target population, and approach to validation. The TASC (Sarason et al., 1960) and CTAS (Wren and Benson, 2004) were specifically developed for children (up to grade 6), whereas the TAI was developed for students high-school age or older (Spielberger, 1980). The methodologies used to develop the WTAS and N&D were, to the best of our knowledge, never reported (and therefore the target population is unknown). The TASC, CTAS, and TAI were validated during their development to ensure that they measured the dimensions they purport to measure. For example, the CTAS was initially composed of 50 items and administered to 230 children; 20 items were subsequently removed due to poor correlation with their intended dimension (either ‘thoughts,’ autonomic reactions, or off-task behaviors), and the remaining 30 items were then administered to 261 students. In this validation sample, the authors found an internal consistency estimate of 0.92 for the total score; 0.76 for the off-task behaviors subscale, 0.82 for the autonomic reactions subscale, and 0.89 for the ‘thoughts’ scale (which included self-critical thoughts, test-related concerns, and test-irrelevant thoughts) (Wren and Benson, 2004). On the other hand, there is no evidence of the validation of the WTAS during its initial development. However, a later study by the developer of the WTAS found that test anxiety reduction techniques administered to 25 college students and 34 5th graders modestly improved grades. The WTAS was administered before and after the anxiety reduction training and the authors found that a decrease in WTAS score significantly correlated with improved grades (*r* = 0.44). Unfortunately, those results were never published in a peer-reviewed process and this statistically significant correlation was found despite the anxiety reduction training not causing a significant change in WTAS scores (Driscoll, 2007). The N&D is a curious case – it is very commonly used by colleges and universities as a screening tool for TA and it is commonly cited in the literature; however, all studies that use the N&D cite “Nist and Diehl, 1990” and various websites where the instrument was found. However, most of those links no longer work, and what appears to be the original website that hosted the instrument (phcc.edu) no longer exists. To the best of our knowledge, neither the developmental process, target population, or validation approach were ever published by the developers of the instrument.

A critical limitation of existing test anxiety instruments is that none directly measure functional impairment, which is a required diagnostic criterion for anxiety disorders in the DSM-5 (APA, 2013). Although the WTAS purports to measure impairment (“memory loss and poor cognitive processing”), the six items related to this domain are subjective questions about the ability to concentrate or remember studied information and do not directly measure changes in test performance due to TA.

Although not necessarily a flaw, all of the previously mentioned TA assessments are based on various psychological models of anxiety and subjectively measure some combination of domains including worry, emotionality, cognitive interference, and physiological overarousal. The benefits of such an approach include the low cost and ease of administration and scoring such assessments as well as allowing the internal construct validity to be readily assessed using factor analysis and related methods. Unfortunately, it cannot control for responder bias nor can it objectively measure functional impairment. In fact, significant response biases based on gender, race, and cultural background have been documented on the CTAS (Aydin, 2019; Nyroos et al., 2015; Wren and Benson, 2004), TAI (Eman et al., 2012; Everson et al., 1991), and TASC (Guida and Ludlow, 1989; Carter et al., 2008) and many studies have failed to find a significant or meaningful effect of TA, as measured by one of these instruments, on academic performance or achievement (Zeidner and Safir, 1988; Chapell et al., 2005; Karatas et al., 2013; Dawood et al., 2016; Cheraghian et al., 2008; Balogun et al., 2017; Zhang and Henderson, 2014). In an effort to overcome these limitations, the current work uses the attentional control theory of anxiety (ACT) as its theoretical basis (one of the most empirically supported neurocognitive models of anxiety) and is a computerized task that specifically measures functional impairment caused by negative performance feedback.

The ACT (Eysenck et al., 2007; Eysenck and Derakshan, 2011), which is an elaboration of the processing efficiency theory, posits that anxiety competes for cognitive resources, which ultimately impairs the efficiency of three executive control functions: the inhibition function, the shifting function, and the updating function. While executive efficiency is impaired, performance may be preserved through compensatory resource allocation; however, when task demands exceed available resources, performance declines.

A substantial body of evidence supports the predictions of the ACT (for review, see Cisler and Koster, 2010; Berggren and Derakshan, 2013; Shi et al., 2019; Ghassemzadeh et al., 2019), and an increasingly large body of neuroimaging and neurophysiology studies are consistent with the predictions of the ACT (for review, see Eysenck et al., 2023). The ACT predicts that anxiety causes increased attention to or loss of attentional control from salient (i.e., threatening) stimuli, resulting in increased resource utilization by the top-down (i.e., task-relevant) attentional system to compensate. Based on this, the operational definition of test anxiety used in developing the Performance Evaluation Anxiety Task (PEAT) was: test anxiety is worry, fear, and loss of attentional control caused by negative performance evaluation (or the threat of a negative performance evaluation), leading to an actual decrease in performance due to competition for cognitive resources.

The goals of the present work were to develop a computerized task that objectively quantifies TA using the framework of the ACT, and then validate this computerized task.

## 2 Methods

### 2.1 Task

**Figure 1:**
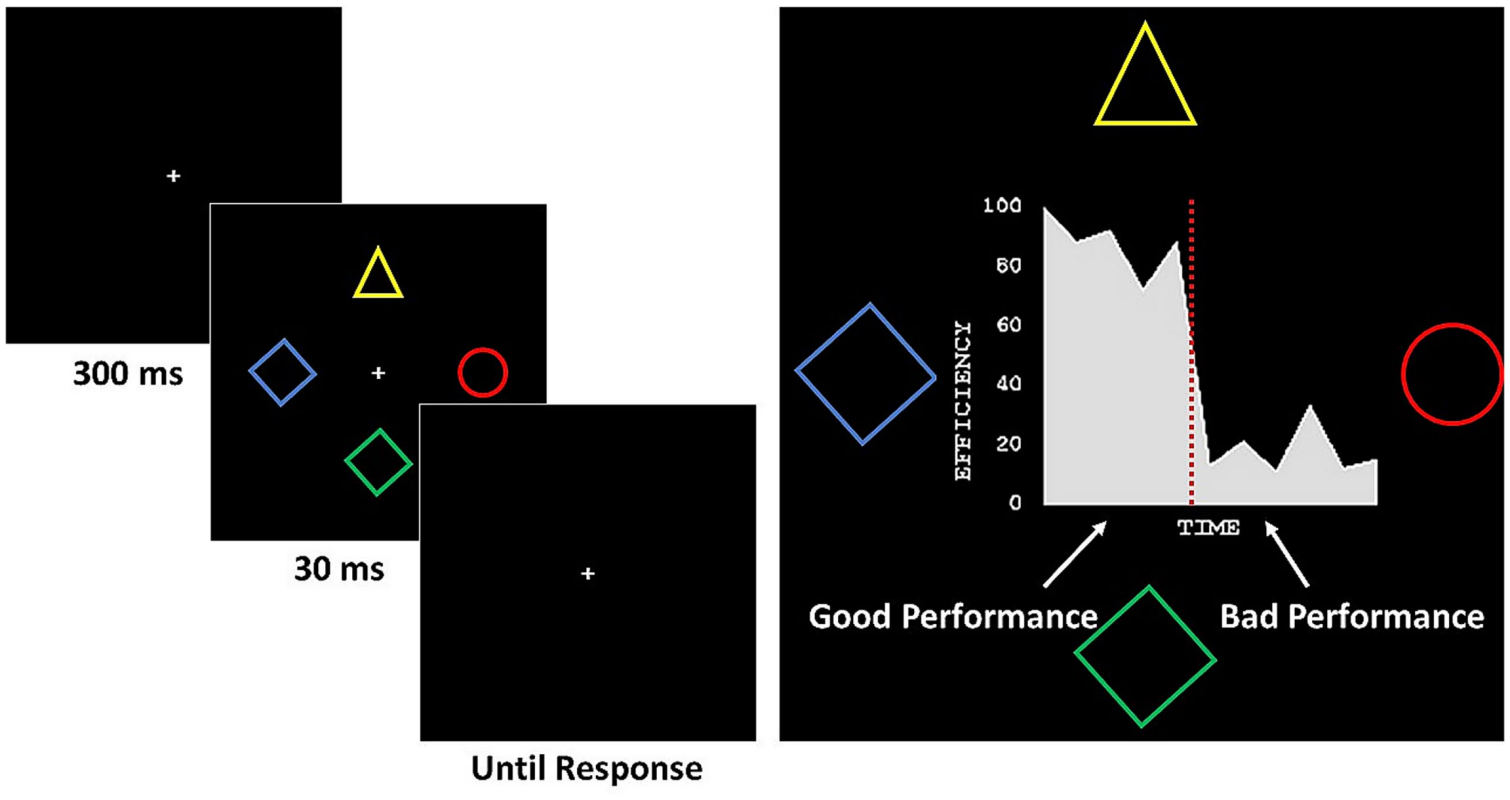
PEAT setup. During the first phase of the task, a central fixation cue is present for 300 ms, followed by four shapes for 30 ms. The participant is to indicate the location of the blue square as quickly and accurately as possible using the arrow keys on the keyboard. The second phase of the task is identical except participants are shown a performance indicator in the center of the screen. The first 25 trials show good performance; the final 25 trials show poor performance. As our operational definition of test anxiety is that HTA individuals will have increased attentional capture or loss of attentional control by a negative performance evaluation, the PEAT score was calculated as the percent change in performance (reaction time / accuracy) during the second phase on the final 25 trials (negative performance evaluation) from the first 25 trials (positive performance evaluation). It would be expected that HTA individuals would have a larger decrease in performance (and thus a more negative PEAT score) than LTA.

The Performance Evaluation Anxiety Task (PEAT) consists of two phases – each phase consists of 50 trials. On each trial, four items are shown for 30 ms around a central fixation point (crosshair), one to the left, one to the right, one above and one below. Each item can be a circle, square, or triangle and could be drawn in red, yellow, blue, or green against a black background. Every trial contains a blue square, which is the target. Participants are instructed to indicate the location of the target using the arrow keys on the keyboard as fast and accurately as possible. Each trial contains a 300 ms intertrial interval. During the first phase of 50 trials, the central visual field consists of only a crosshair. During the second phase of 50 trials, participants are told that the central region will show a performance indicator that compares their current performance relative to their performance during the first phase. Unbeknownst to the participant, the performance indicator shown is predetermined – the first 25 trials show high performance (>80% efficiency) whereas the final 25 trials show poor performance (<30% efficiency). The central fixation point (during the first phase) and the performance indicator (during the second phase) are shown continuously. The PEAT was designed using Paradigm Experiments (formerly by Perception Research Systems, Inc.). The overall task setup is shown in Figure 1.

As the items in each trial are only shown for 30 ms and the actual task is peripheral to the performance indicator, an increase in attention to the performance indicator and/or a loss of attentional control to the task will cause a decrease in actual task performance. The ACT predicts that HTA will have increased attention to the performance indicator and/or loss of attentional control to the task specifically while being given negative performance feedback, whereas the attentional dynamics should remain constant across the task in LTA. Therefore, the PEAT score was calculated as the change in performance (reaction time / proportion of trials answered correctly) during the trials showing negative feedback relative to positive performance. In effect, the first phase of the PEAT served as practice trials and provides pretext for the presence of the performance indicator in the second phase of the task.

### 2.2 Validation approach

The PEAT was validated using four complementary approaches designed to assess physiological, attentional, executive, and discriminant dimensions of evaluation-related vulnerability. First, cortisol reactivity during performance evaluation was examined. Second, attentional engagement, disengagement, and neural markers of cognitive control were assessed in a dot-probe paradigm. Third, feedback-induced executive control disruption was evaluated in a go/no-go task. Finally, discriminant validity was tested by examining associations with ADHD diagnosis and medication use.

Once the best cutoff score had been determined, it was expected that neurobehavioral responses predicted by the ACT should be evident in HTA but not LTA. According to the ACT, anxious individuals should attend to threatening information more or have attentional control disrupted more from threatening information than non-anxious individuals, or both. Critically, what is considered threatening should be domain specific (HTA should consider words such as “failure” more threatening than the word “murder,” the opposite of what would be expected with someone with generalized anxiety disorder). Therefore, in one study, participants completed a dot-probe task (DPT) that included threatening words from two categories: test-specific threat (TST) and general threat (GT). During the DPT, participants had electroencephalographic (EEG) data collected to measure a number of neural indices of attentional bias and loss of attentional control along with the standard behavioral indices measured by the DPT. It was expected that HTA would have a larger attentional bias towards TST than GT whereas LTA would have the opposite pattern. Because the DPT included trials containing only neutral words (no threat), attentional engagement and disengagement scores could also be calculated (measures of attentional capture by threat and attentional disengagement from threat) (Loomis et al., 2023; Koster et al., 2006). It was predicted that HTA would have higher engagement and lower disengagement scores to TST than GT whereas LTA would have the opposite. On EEG, the N2pc wave, frontal midline theta power (FMθ), frontocentral theta/beta ratio (TBR), posterior theta power (Pθ), and posterior alpha power (Pα) were measured. The N2pc wave is an event-related potential component that indexes the automatic allocation of spatial attention contralaterally (i.e., if attention is allocated to the left hemifield, there is a negative deflection on the right posterior cortex). It is commonly reported that anxious individuals have a larger N2pc wave to threat (Wieser et al., 2018; Reutter et al., 2017; Berggren and Eimer, 2021; Salahub and Emrich, 2020), although it is not universally seen and is considered unreliable by some authors (Hu et al., 2023; Kappenman et al., 2021; Kappenman et al., 2014). It was predicted that HTA would have a larger N2pc to TST than GT whereas LTA would have the opposite. FMθ is an index of a general need for cognitive control (Cavanagh and Frank, 2014) and is increased in anxiety (Shalamberidze et al., 2025; Cavanagh et al., 2017; McLoughlin et al., 2022). It was predicted that HTA would have an elevated FMθ to TST relative to GT whereas LTA would have the opposite. TBR is an index of attentional control, with an increase in TBR indicating a loss of attentional control or increased effort to control attention (Putman et al., 2014). It was expected that HTA would have an elevated TBR to TST relative to GT whereas LTA would have the opposite. Pθ is a marker of enhanced processing of threatening or emotional stimuli (Zhong et al., 2023; Knyazev et al., 2009), and it was expected that HTA would have a higher Pθ to TST than GT, the opposite of LTA. Finally, Pα is a marker of cortical inhibition or reduced processing of stimuli (Klimesch, 2012; Klimesch et al., 2007; Foxe and Snyder, 2011). Anxious individuals have been shown to have lower Pα, particularly to threat (suggesting increased processing) (Mirifar et al., 2025; Roxburgh et al., 2023). Therefore, it was expected that HTA would have a smaller increase in Pα than LTA to both threat types, but HTA would have a lower Pα to TST than GT and LTA would have the opposite.

It would also be expected that PEAT scores would predict executive control dysfunction consistent with the ACT in a task unrelated to the PEAT. Therefore, participants classified as either LTA or HTA using the PEAT completed a modified go/no-go task. The modification to the classic version of this task was that participants did the task twice; once without performance feedback and again with negative performance feedback. According to the ACT, highly test anxious participants should have disruption of the inhibitory and updating functions of executive control specifically during the negative performance feedback. Therefore, we hypothesized that HTA participants would exhibit a larger number of commission errors (indicative of inhibitory failure) (Wright et al., 2014) as well as reduced post-error slowing (indicative of updating disruption) (Dutilh et al., 2012) relative to LTA during the second phase of the task.

Finally, because the PEAT was developed based on the ACT and is measuring the loss of attentional control, it was important to ensure that the PEAT was measuring loss of attentional control specifically due to negative performance feedback and not poor attentional control due to other reasons. Therefore, in the final study, participants were asked about previous diagnoses of attention-deficit/hyperactivity disorder (ADHD) as well as current medicine being used to treat ADHD. If the PEAT was measuring poor attentional control unrelated to TA, it would be expected that the PEAT would classify a higher proportion of participants with ADHD as HTA.

### 2.3 Validation methods

The PEAT was administered to a total of 573 participants (414 undergraduate students and 140 graduate students; 19 students did not specify their level). Fifty-six were involved in the dot-probe task study, 207 were involved in the cortisol assay study, 39 were involved in the go/no-go study, 75 were involved in the ADHD/TA discrimination study, and the remaining 196 only took the PEAT to help determine the normal distribution of scores or were part of other studies not reported here. All studies were approved by the Institutional Review Board of Mississippi College, and all participants provided written informed consent.

Participants that were recruited into the cortisol study were excluded if they had taken any medication containing stimulant properties (i.e., amphetamine) or excessive intake of alcohol or caffeine within 24 h prior, had eaten food, chewed gum, or brushed teeth within one hour, or if they had engaged in strenuous physical activity within 48 h prior to the experiment. Because of the diurnal pattern of cortisol production, all experiments were conducted between 1 p.m. and 4 p.m. Participants were given two Salivette® Cortisol vials (Sarstedt), one for a baseline cortisol measurement and one to measure cortisol after the task. At each collection, a synthetic swab was placed between the cheek and bottom row of teeth for 2 min, then placed back into the vial. The participant then completed the PEAT; at the completion of the PEAT, the program began a 15-min countdown, during which time the participants were instructed to remain seated and refrain from speaking to other participants or the researcher. Following the 15-min waiting period, participants were instructed to repeat the saliva collection with the second tube. This 15-min waiting period before resampling was chosen as the salivary cortisol response to acute stress in laboratory settings peaks between 10 and 30 min following the event (Foley and Kirschbaum, 2010). Saliva samples were centrifuged at 1000 x g for two minutes (to extract cell-free saliva from the swab) and then stored at −20° C until the cortisol assay was performed (within 4 weeks of collection). The cortisol assay used was Parameter™ Cortisol Assay, a commercially available ELISA kit (R&D Systems) using the manufacturer’s instructions. All samples and standards were measured in duplicate.

For the DPT study, potential participants were first screened for test anxiety using the PEAT and an equal number of low- and high-test anxious participants were invited to participate. Participants were first connected to a 9-lead EEG (Advanced Brain Monitoring X10). EEG data were collected at 256 Hz via iMotions software (Copenhagen, Denmark). Resistance on all electrodes was kept <40 kΩ (as recommended by the manufacturer) with EEG data referenced to linked mastoids. The DPT was programmed using Paradigm software and administered using Paradigm Player (version 2.1.0) and consisted of 150 trials: 50 neutral-neutral pairs, 50 neutral-TST pairs, and 50 neutral-GT pairs (Table 1 shows the words used for each trial type). Words in the TST category were chosen for their relevance to testing, performance evaluation, or the teaching environment as well as their valence and arousal ratings from normative databases (Bradley and Lang, 1999; Warriner et al., 2013). Words in the GT category were chosen for their relevance to violence, disgust, or traumatic events as well as their valence and arousal ratings (Bradley and Lang, 1999; Warriner et al., 2013). Neutral words were chosen to match the length of the threatening words as well as their valence and arousal ratings (Bradley and Lang, 1999; Warriner et al., 2013). Each trial showed a central fixation cross for 3,000 ms, followed by a word pair for 1,000 ms (one to the left and one to the right of the fixation cross, matched for length). One of the words is then replaced by the probe (the symbol “*”). The participant is instructed to indicate, using the arrow keys on the keyboard, whether the probe was shown on the left or right as quickly and accurately as possible. Probes were presented equally on the left and right sides, and probes replaced the threatening or neutral word with equal frequency. EEG data were analyzed using Python scripts (available at https://doi.org/10.7910/DVN/NXDZMA). Briefly, the continuous EEG was notch filtered between 55 and 65 Hz, bandpass filtered between 0.2 and 50 Hz, and epoched from −200 ms prior to the onset of word presentation to 1,000 ms after the onset of word presentation (−200 to 0 ms was used as baseline). Data epoched around the probe presentation is not part of this study. Independent components analysis was performed to identify and remove non-EEG components (primarily eyeblinks and ocular movement components). Individual epochs were automatically rejected using peak-to-peak amplitude (>200 μV), maximum voltage amplitude (>100 μV), and root mean square robust z-threshold (>5). For N2pc amplitude, data from the left parietal lobe (P3) and right parietal lobe (P4) were used to compile event-related potentials (ERPs) when the threatening word was presented ipsilateral or contralateral to these sites. The N2pc amplitude was taken to be the 20 ms average of the difference wave (ipsilateral – contralateral ERPs) that was most negative between 200 and 350 ms post-stimulus onset. For time-frequency analyses, complex Morlet wavelets were used to calculate power separately for theta (4–7 Hz), alpha (8–12 Hz), and beta (13–30 Hz) frequency ranges; frequencies were sampled at ~0.5 Hz resolution within each band and a fixed wavelet width of 5 cycles was used for all frequencies (capped when necessary to ensure their temporal extent did not exceed epoch length). Power estimates were baseline corrected from −200 to 0 ms with ratio normalization. Analyses focused on a 300–500 ms post-stimulus window based on previous findings that FMθ peaks ~300–500 ms (Cohen, 2014; Cavanagh and Frank, 2014; Darna et al., 2025) as does sensory suppression via alpha-band synchronization (Foxe and Snyder, 2011; Bacigalupo and Luck, 2022).

**Table 1:** List of words used in the dot-probe task.

Neutral words		GT words	TST words
ACTOR	GOOGLE	ALARMED	ASSESSMENT
AFFIRMATION	JUMP	ATTACKS	AVERAGE
AISLE	KITCHEN	BANKRUPTCY	CHEATS
BABY	LIGHT	BLOOD	DIFFICULT
BALLOT	MIDDLE	CANCER	ERRORS
BAND	MUSEUM	DANGER	EVALUATION
BOAT	NAME	DARK	EXAM
BOOK	NIGHTS	DEFECT	FAILURE
BRACELETS	OPENING	DEMISE	FINALS
BREAD	ORANGE	DESPERATE	GRADES
FLOWERS	PINK	STRESS	HARD
CHICKEN	PLANET	EXPIRE	HIGH
CITRONELLA	RAINBOWS	FEAR	LATE
CONTESTANTS	RELIEF	GORE	LOW
COPPER	ROSE	HARMS	MARKS
COZY	SALTWATER	HAZARD	MISTAKE
CURTAIN	STRAWBERRY	HOPELESS	PASS
DAYS	SUMMER	HUMILIATE	PERFORMANCE
DECADE	SUN	LOSS	PRESSURE
DIGITAL	TELEVISION	PAIN	QUIZ
DOOR	THANKS	PANICKY	SCORE
DRAW	TOE	SAD	TEACHER
EARS	UMBRELLA	SICKLY	TEST
FABRIC	URBAN	SUICIDE	TIME
GIGGLES	WATERPROOF	TENSE	WRONG
GIRL	YOUNG		

Each threatening word was used twice; once on the left side and once on the right side. Neutral items were also used multiple times.

Similar to the DPT, for the go/no-go study, potential participants were first screened for test anxiety using the PEAT and an equal number of low- and high-test anxious participants were invited to participate. Participants were connected to the same EEG equipment as described above (however, the EEG data collected is not part of this study). Both phases of the go/no-go task consisted of 1,000 trials. Each trial began with a central fixation cross shown for 500 ms, followed by the presentation of five numbers (0–9). The numbers could be shown in red or blue against a white background. Any trial containing a red 7 was a no-go trial, in which case the participant was to press the ‘j’ key on the keyboard. Otherwise, the participant was to press the space bar. Eighty percent of the trials were ‘go’ trials, 20% were no-go trials. The numbers were shown until the participant responded, up to 500 ms. Following the response (or 500 ms), a screen with a central fixation cross was shown for 1,000 ms. The second phase of the task was identical except participants were told that they would be shown a performance indicator in the upper right corner of the screen and that their performance was being calculated relative to a standard population. However, all participants were shown the same, negative performance (<25th percentile). Participants were given a self-paced break between the first and second phases as well as halfway through each phase.

Participants that were recruited into the ADHD/TA discrimination study first completed a computerized survey that asked whether there was a previous diagnosis of attention-deficit disorder (ADD) or ADHD from a clinician (psychologist, psychiatrist, pediatrician, nurse practitioner, etc.) and, regardless of a diagnosis, if they were currently taking any medication to treat ADHD (and if so, which medicine). Following this, participants completed the PEAT.

### 2.4 Statistical analyses

Valence and arousal ratings between neutral and GT or TST items were compared using two sample t-tests assuming unequal variance (Welch’s t-test). In the dot-probe task, attentional bias was calculated by subtracting the average reaction time (RT) on congruent trials from the average RT on incongruent trials. Attentional engagement was calculated by subtracting the average RT on congruent trials from the average RT on trials containing two neutral items and attentional disengagement was calculated by subtracting the average RT on trials containing two neutral items from the average RT on incongruent trials. To compare attentional biases, engagement, and disengagement scores to TST and GT between HTA and LTA, two-way analyses of variance (2-way ANOVA) were used. Because three different behavioral parameters were analyzed separately using 2-way ANOVA, the Benjamini-Hochberg procedure to control the false discovery rate (FDR correction) was applied to the *p*-values of the main effect of level of test anxiety, the p-values of the main effect of threat type, and the p-values for the interaction term. To determine if changes in FMθ, TBR, Pθ, and P*α* to TST and GT varied as a function of test-anxiety group, 2-way ANOVAs were used on baseline-normalized power values with the primary contrast of interest being the interaction between factors. For FMθ and TBR, power from electrodes Fz and Cz were collapsed; for Pθ and P*α*, power from electrodes P3, P4, and POz were collapsed. Because four different EEG parameters were analyzed separately using 2-way ANOVA, the same FDR correction procedure as above was used on the p-values of the interaction term. A sensitivity power analysis indicated that, with *N* = 55, α = 0.05, and power = 0.80, the study was powered to detect interaction effects of approximately *f* = 0.38 (*η_p_^2^* = 0.12). In the go/no-go task, commission error rate was calculated as the total number of incorrect button-presses on no-go trials divided by the total number of no-go trials; post-error slowing (PES) was calculated as the mean RT following a correct response subtracted from the mean RT following an error. An ‘evaluation reactivity index’ (ERI) was also computed to capture the combination of both commission error rate and PES. The ERI was calculated using z-scored values of commission error rate minus z-scored values of PES. The value of PES was subtracted from commission error rate as a decrease in PES reflects dysregulation of the updating function of executive control. To determine if changes in commission error rate, PES, and ERI varied as a function of test anxiety group based on feedback, 2-way ANOVAs were used. A sensitivity power analysis indicated that, with *N* = 39, α = 0.05, and power = 0.80, the study was powered to detect interaction effects of approximately *f* = 0.45 (*η_p_^2^* = 0.17). To compare the change in salivary cortisol between HTA and LTA, Welch’s t-test was used. A sensitivity power analysis indicated that, with n = 165 and n = 40, α = 0.05, and power = 0.80, the study was powered to detect group differences of approximately d = 0.49 or larger. To determine if there was a relationship between being classified as either HTA or LTA on the PEAT and having a diagnosis of ADHD or using medicine for ADHD, Fisher’s exact test was used. A sensitivity analysis for a 2×2 contingency table indicated that, given the sample sizes, α = 0.05, and power = 0.80, the study was powered to detect a difference in proportions of approximately 0.20 between groups. All data analysis was performed using GraphPad Prism (version 10.0.1). All data are presented as mean +/− SEM unless otherwise indicated. In all cases, *p* < 0.05 was considered statistically significant.

## 3 Results

### 3.1 Distribution of PEAT scores and score cutoff determination

**Figure 2:**
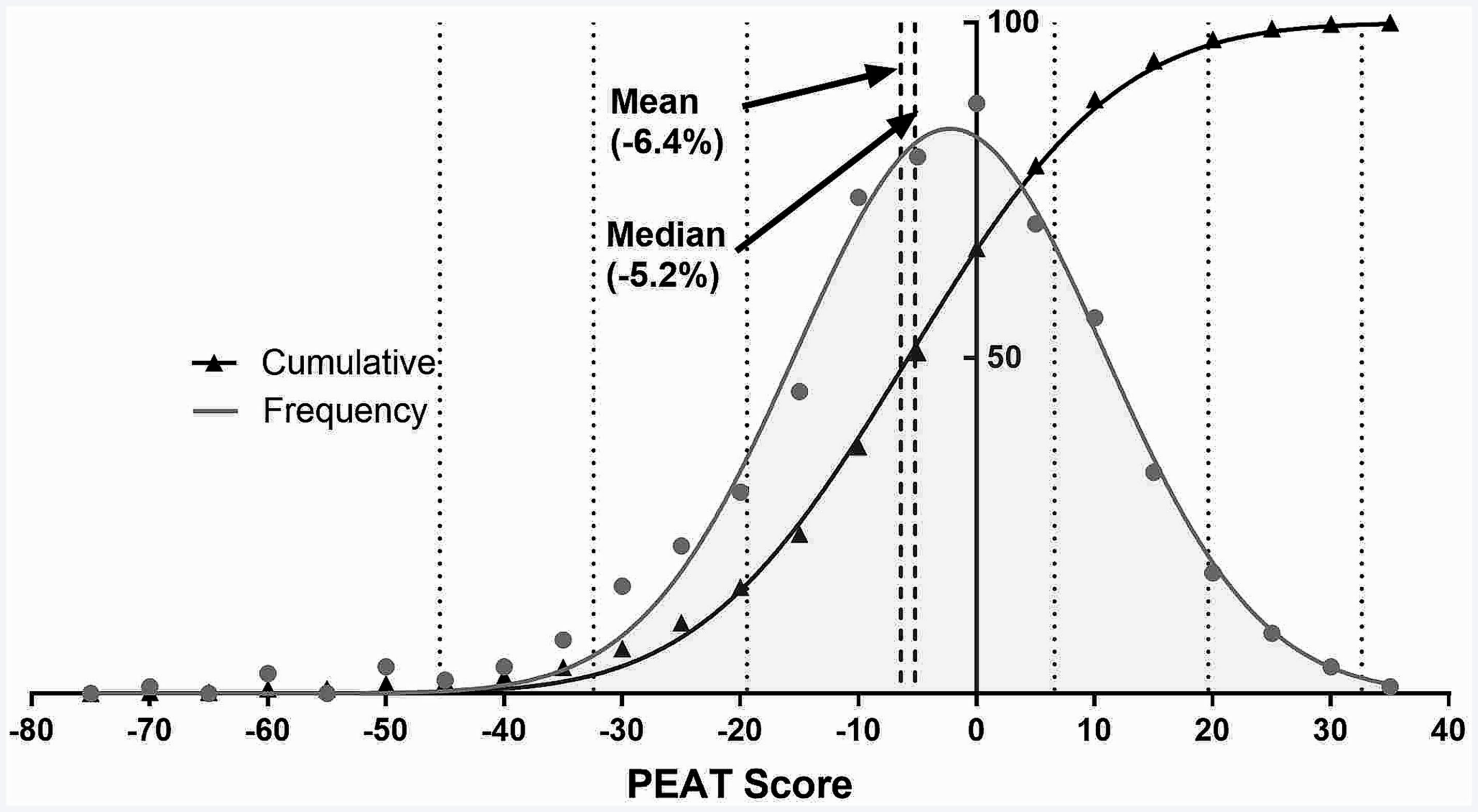
Cumulative and frequency distribution of PEAT scores in 573 participants. These data were fit to least squares Gaussian curves. Vertical dashed lines indicate the mean and median of the curve-fitted frequency data, vertical dotted lines indicate standard deviations away from the mean.

Each participant’s score was calculated as the percent change in performance (RT divided by accuracy) from the first 25 trials of the second phase (during positive feedback) to the final 25 trials (during negative feedback). The mean and median of the raw score distribution were −6.4% and −5.2%, respectively. As expected, the data had a normal Gaussian distribution, so the distribution of scores were fit to a Gaussian (least squares) curve. The Gaussian curve fit to the frequency data had an R^2^ = 0.9872, a mean of −2.23% and a standard deviation of 13.01. The Gaussian curve fit to the cumulative frequency data had an R^2^ = 0.9991, a mean of −5.79%, and a standard deviation of 13.9. These data are shown in Figure 2.

The frequency distribution data provided a starting point to determine what score should be considered HTA. It was initially assumed that if estimates of TA in students are correct (15–50% of students), a cutoff of one standard deviation below the mean or lower would be a good starting point as that would encompass approximately 15% of the population.

### 3.2 Change in cortisol

**Figure 3:**
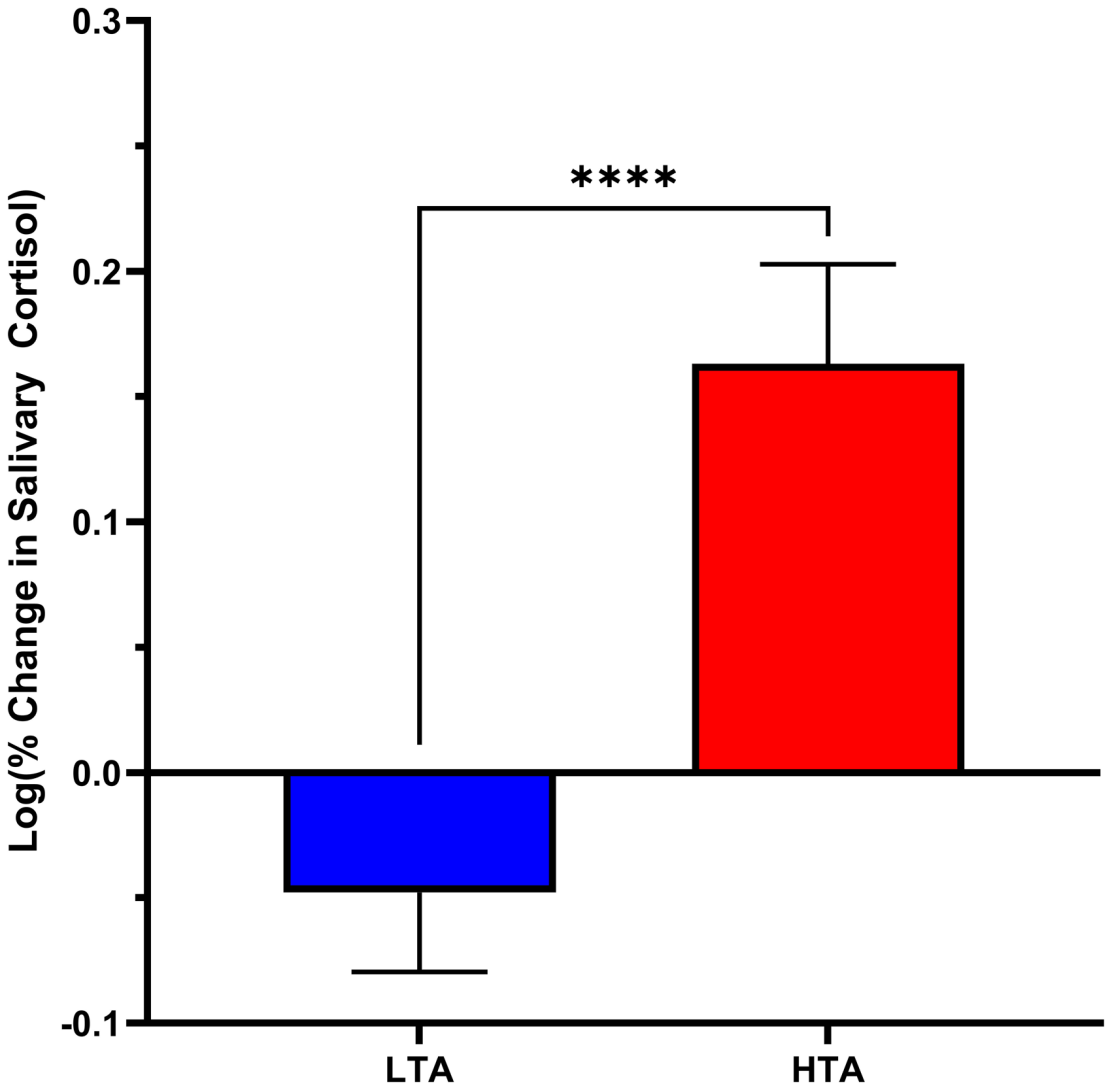
Log-transformed percent change in salivary cortisol between LTA and HTA (defined as a PEAT score of −15% or more). Data were log transformed due to non-normality.

Two hundred and seven participants provided saliva samples before (baseline) and after completing the PEAT. Data were calculated as percent change in salivary cortisol after the PEAT relative to baseline; data were then log transformed as the distribution was significantly non-normal (mean = 24.6, median = 3.09, skew = 1.2, kurtosis = 1.16; Kolmogorov–Smirnov D = 0.12, *p* = 0.04). Because of negative values, a constant (72) was added to all values before log transformation; after log transformation, the antilog of the constant (1.857) was removed from all values. Following transformation, two data points were determined to be outliers (>4 standard deviations from the mean) and were removed. After transformation and outlier removal, data were normal (mean = 1.85, median = 1.89, skew = −0.68, kurtosis = 0.41, K-S D = 0.074, *p* > 0.05). Participants were divided into LTA or HTA groups using a PEAT score of −15% or less to indicate HTA (as suggested by the normal distribution and estimate of TA prevalence data). Based on this cutoff, 40 participants (19.5%) were classified as HTA, 165 were classified as LTA. There was a significant difference in the log change of salivary cortisol between LTA (−0.048 +/− 0.032) and HTA (0.163 +/− 0.040) with Welch’s t(95.34) = 4.151, *p* < 0.0001, d = 0.621. Repeating the analysis using other potential cutoffs on the PEAT (−10% or more, −20% or more, etc.) did not improve (or worsened) the separation between groups. These data are shown in Figure 3.

Candidate cutoff scores were initially identified based on the score distribution and published prevalence estimates of test anxiety. Cortisol reactivity was then used as an external physiological criterion to evaluate whether these candidate thresholds differentiated individuals showing heightened stress responses during evaluation. The −15% threshold demonstrated the strongest physiological differentiation while remaining consistent with expected prevalence estimates and was therefore retained for subsequent analyses.

### 3.3 Dot-probe task and EEG parameters

As expected, the mean valence ratings between neutral (6.25 +/− 0.13) and GT items (2.61 +/− 0.18) were significantly different (Welch’s t(49.57) = 16.30, *p* < 0.0001, d = 3.90) as were the mean arousal ratings between neutral (4.08 +/− 0.10) and GT items (5.68 +/− 0.25) (Welch’s t(29.74), t = 5.89, *p* < 0.0001, d = 1.41). Also as expected, the mean valence ratings between neutral (6.25 +/− 0.13) and TST items (4.47 +/− 0.24) were significantly different (Welch’s t(37.94) = 6.40, *p* < 0.0001, d = 1.53) as were the mean arousal ratings between neutral (4.08 +/− 0.1) and TST items (4.73 +/− 0.20) (Welch’s t(33.82) = 2.88, *p* = 0.0034, d = 0.69). Of note, the mean valence ratings between GT and TST were also significantly different (Welch’s t(42.17) = 6.22, *p* < 0.0001, d = 1.49) as were the mean arousal ratings between GT and TST items (Welch’s t(43.7) = 2.96, *p* = 0.0025, d = 0.71), with the TST items having a higher valence and being less arousing than the GT items. This likely reflects the non-threatening nature of words such as “performance” (valence = 6.2), “teacher” (valence = 5.68), and “time” (valence = 5.15) to the general population, however threatening they may be to HTA individuals.

**Figure 4:**
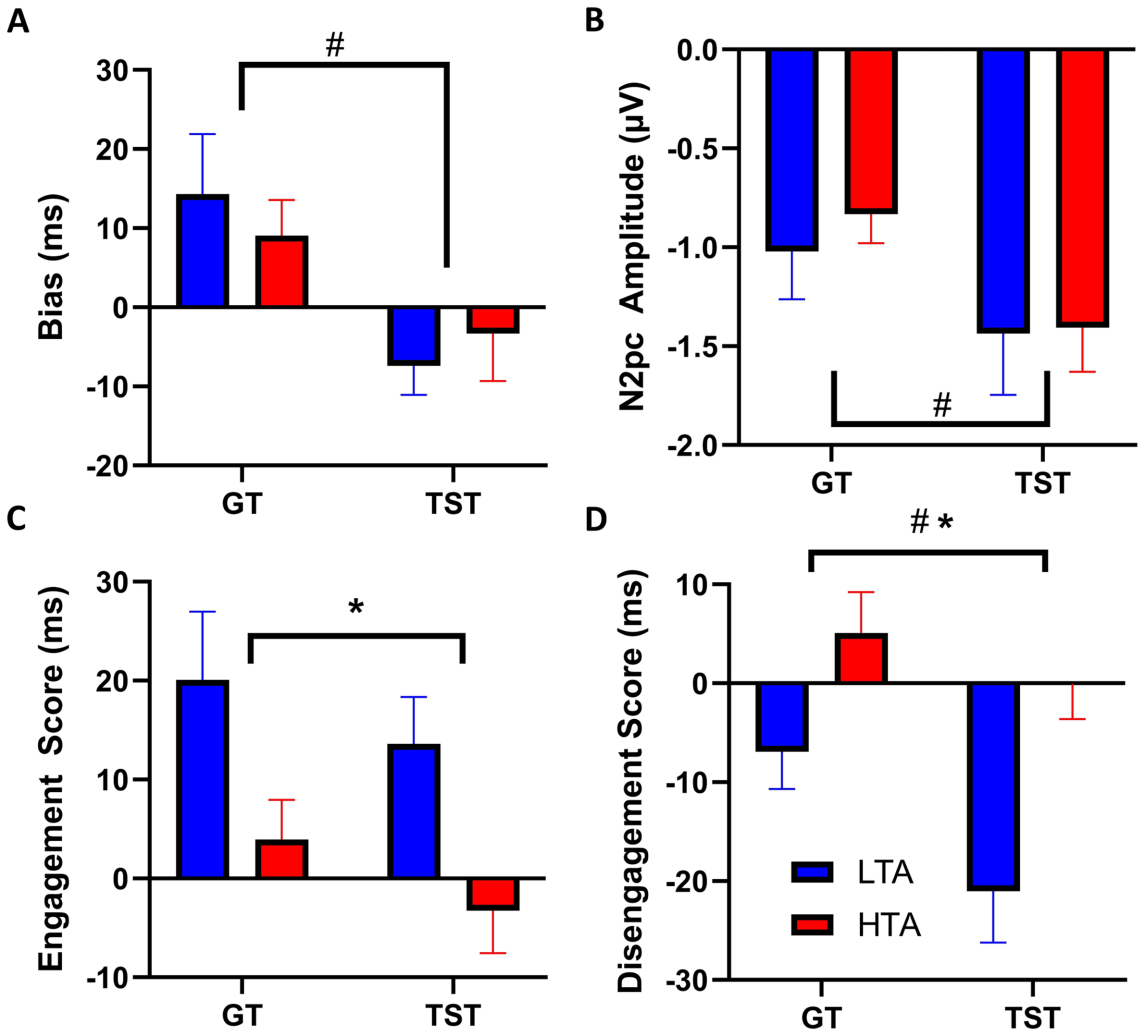
Attentional bias, N2pc amplitude, engagement, and disengagement scores to general threat and test-specific threat. **(A)** Attentional bias scores. Bias towards GT was higher than TST regardless of level of test anxiety. **(B)** N2pc peak amplitude. N2pc amplitude was larger (more negative) to TST items than GT items regardless of level of test anxiety. **(C)** Attentional engagement scores. LTA had significantly higher engagement than HTA regardless of threat type. **(D)** Attentional disengagement scores. LTA had significantly more negative scores (indicating more rapid disengagement) than HTA, and disengagement scores were more negative for TST items than GT items. * Indicates significant difference between LTA and HTA, # indicates significant difference between GT and TST.

There were 56 participants recruited into the DPT study; however, EEG data from one participant was unusable due to poor signal quality, leaving 30 LTA and 25 HTA in the final analyses (using a PEAT cutoff score of −15% or less). A 2-way analysis of variance (ANOVA) was conducted to determine if attentional bias towards (or away from) threat varied due to level of test anxiety or threat type. There was no main effect of level of test anxiety [*F*(1, 53) = 0.011, *p* > 0.05, *η_p_^2^* = 0.0002], although there was a main effect of threat type [*F*(1, 53) = 8.4, *p* = 0.005, *η_p_^2^* = 0.137] which survived FDR correction; the interaction term was not significant [*F*(1, 53) = 0.634, *p* > 0.05, *η_p_^2^* = 0.012]. Participants had a higher bias towards general threat than test-specific threat. These data are shown in Figure 4 (panel A). The same analysis was conducted on attentional engagement scores. There was a significant main effect of level of test anxiety [*F*(1, 53) = 7.08, *p* = 0.01, *η_p_^2^* = 0.118], which survived FDR correction, but not threat type [*F*(1, 53) = 2.62, *p* > 0.05, *η_p_^2^* = 0.05]; the interaction term was not significant [*F*(1, 53) = 0.007, *p* > 0.05, *η_p_^2^* = 0.0001]. LTA had significantly higher engagement scores than HTA. These data are shown in Figure 4 (panel C). The same analysis was conducted on attentional disengagement scores. There was a significant main effect of level of test anxiety [*F*(1, 53) = 12.42, *p* = 0.0009, *η_p_^2^* = 0.190] as well as threat type [*F*(1, 53) = 6.11, *p* = 0.016, *η_p_^2^* = 0.103], both of which survived FDR correction; the interaction term was not significant [*F*(1, 53) = 1.32, *p* > 0.05, *η_p_^2^* = 0.024]. Overall, disengagement scores were lower in LTA than HTA, and were lower for TST items than GT items. These data are shown in Figure 4 (panel D).

The same analysis was conducted on N2pc amplitude. There was no main effect of level of test anxiety [F(1, 53) = 0.189, *p* > 0.05, *η_p_^2^* = 0.004] although there was a main effect of threat type [F(1, 53) = 4.062, *p* = 0.049, *η_p_^2^* = 0.071] such that N2pc amplitudes to TST items were of larger magnitude than to GT items. However, there was not a significant interaction between factors [F(1, 53) = 0.104, *p* > 0.05, *η_p_^2^* = 0.002], which was an unexpected finding. These data are shown in Figure 4 (panel B).

**Figure 5:**
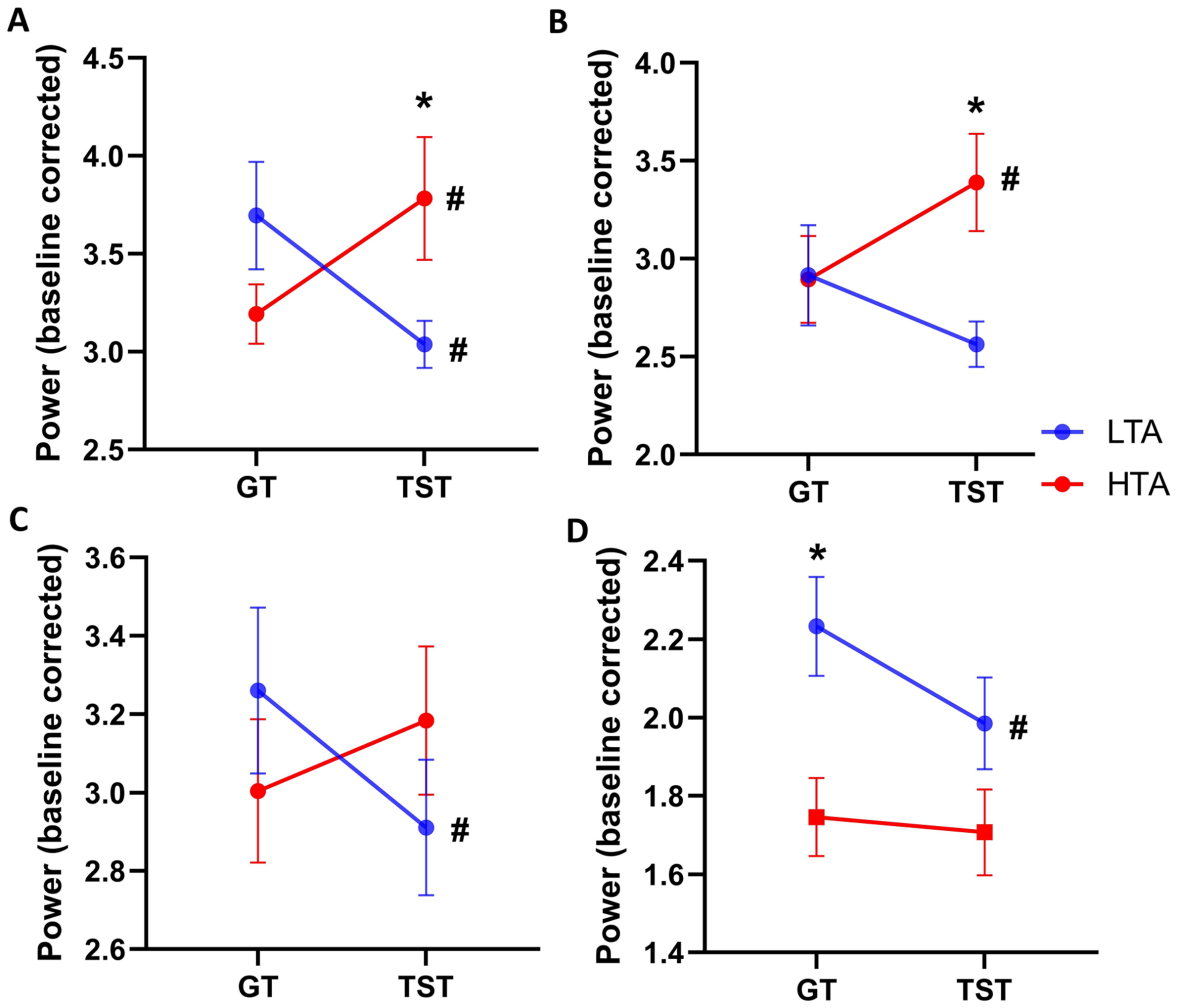
Midfrontal theta power (FMθ), theta/beta ratio (TBR), parietal theta power (Pθ), and parietal alpha power (Pα) changes (relative to baseline) 300–500 ms post-stimulus. **(A)** Shows FMθ power. LTA and HTA did not differ on general threat trials; however, HTA had a significant increase and LTA had a significant decrease in power relative to GT. **(B)** Shows TBR. Similar to FMθ, LTA and HTA did not differ on GT trials; on TST trials, HTA had a significant increase and LTA had a significant decrease in TBR relative to GT. **(C)** Shows Pθ power. LTA had significantly higher power on GT trials compared to TST trials; there were no significant differences between trial types in HTA. **(D)** Shows Pα power. There were no differences between GT and TST trials in HTA; however, LTA had significantly higher power on GT trials compared to TST trials. * Indicates significant difference between LTA and HTA on that threat type. # indicates significant difference between GT and TST in that group.

Four separate 2-way ANOVAs (with FDR correction) were conducted to determine if FMθ, TBR, Pθ, or Pα varied due to level of test anxiety or threat type; however, the interaction term was the *a priori* contrast of interest. For FMθ, there was no main effect of level of test anxiety [F(1, 53) = 0.214, *p* > 0.05, *η_p_^2^* = 0.004] or threat type [F(1, 53) = 0.032, *p* > 0.05, *η_p_^2^* = 0.0006]. Critically, there was a significant interaction between factors [F(1, 53) = 10.98, *p* = 0.0017, *η_p_^2^* = 0.172] which survived FDR correction. Post-hoc analyses showed that for LTA, FMθ was significantly higher for GT items than TST items, whereas for HTA, FMθ was significantly higher for TST items than GT items. Also, HTA had significantly higher FMθ on TST items than LTA. These data are shown in Figure 5 (panel A). For TBR, there was no main effect of level of test anxiety [F(1, 53) = 2.348, *p* > 0.05, *η_p_^2^* = 0.042] or threat type [F(1, 53) = 0.209, *p* > 0.05, *η_p_^2^* = 0.004]. However, there was a significant interaction between factors [F(1, 53) = 7.386, *p* = 0.0089, *η_p_^2^* = 0.122] which survived FDR correction. Post-hoc analyses showed that for HTA, TBR was significantly higher for TST items than GT items. On TST items, HTA had significantly higher TBR than LTA. These data are shown in Figure 5 (panel B). Similarly to FMθ and TBR, there was no main effect on Pθ due to level of test anxiety [F(1, 53) = 0.0011, *p* > 0.05, *η_p_^2^* = 0.00002] or type of threat [F(1, 53) = 0.591, *p* > 0.05, *η_p_^2^* = 0.011], but there was a significant interaction between factors [F(1, 53) = 5.715, *p* = 0.02, *η_p_^2^* = 0.097] which survived FDR correction. Post-hoc analyses showed that for LTA, Pθ was higher on GT items than TST items. These data are shown in Figure 5 (panel C). Unexpectedly for Pα, there was a main effect of level of test anxiety [F(1, 53) = 7.281, *p* = 0.0093, *η_p_^2^* = 0.121] as well as a trend for type of threat [F(1, 53) = 3.029, *p* = 0.087, *η_p_^2^* = 0.054]. However, the planned comparison (the interaction term) was not significant [F(1, 53) = 1.61, *p* = 0.21, *η_p_^2^* = 0.029]. Post-hoc analyses found that LTA have higher Pα on GT trials than HTA and higher Pα on GT trials than TST trials. These data are shown in Figure 5 (panel D).

### 3.4 Executive control dysregulation in the go/no-go task

**Figure 6:**
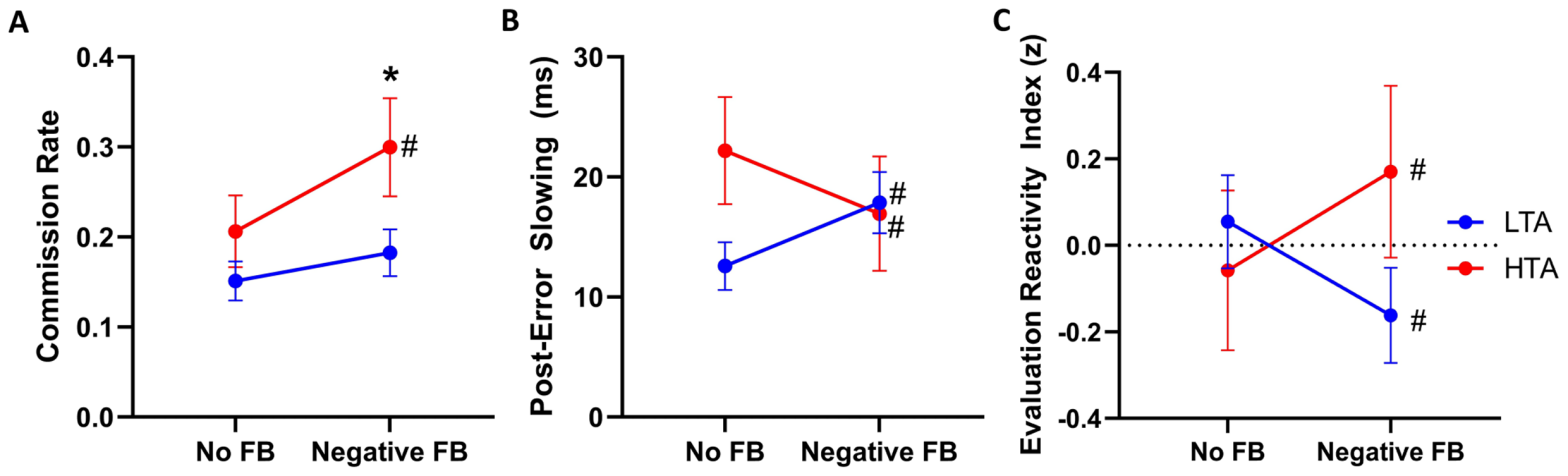
Behavioral measures of executive control regulation in LTA and HTA on a go/no-go task with and without negative feedback. Panel A shows the commission error rate. There were no differences between LTA and HTA in the no feedback condition; however, during negative feedback, HTA had a significant increase in commission rate, which was higher than LTA. Panel B shows post-error slowing (PES). Both LTA and HTA had a significant change from the no feedback condition to the negative feedback condition; however, these significant changes were in opposite directions (LTA had an increase in PES whereas HTA had a decrease in PES). Panel C shows the evaluation reactivity index (ERI), which combines the z-score normalized commission error rate and the z-score normalized PES into a single value, with higher scores indicating decreased executive control inhibitory function, updating function, or both. LTA and HTA had similar ERI scores in the no feedback condition. Both LTA and HTA had a significant change in the negative feedback condition, although in opposite directions (LTA had a significant decrease whereas HTA had a significant increase). * Indicates significant difference between LTA and HTA on that feedback type. # Indicates significant difference between no feedback and negative feedback in that group.

There were 39 participants (19 HTA using a PEAT cutoff score of −15% or less) recruited into the go/no-go study. A 2-way analysis of variance (ANOVA) was conducted to determine if commission error rate varied due to level of test anxiety or feedback. There was a trend towards a significant main effect of level of test anxiety [*F*(1, 37) = 3.021, *p* = 0.09, *η_p_^2^* = 0.076] and a significant main effect of feedback [*F*(1, 37) = 12.52, *p* = 0.001, *η_p_^2^* = 0.253]. The interaction between factors also trended towards significance [F(1, 37) = 3.09, *p* = 0.087, *η_p_^2^* = 0.077]. Post-hoc analyses found that HTA had a higher commission error rate than LTA during negative feedback, and that HTA had a significant increase in commission error rate during feedback relative to the no feedback condition. These data are shown in Figure 6 (panel A). The same analysis was conducted on PES. There was no main effect of level of test anxiety [F(1, 37) = 0.84, *p* > 0.05, *η_p_^2^* = 0.022] or feedback [F(1, 37) < 0.001, *p* > 0.05, *η_p_^2^* < 0.00001]; however, there was a significant interaction between factors [F(1, 37) = 8.55, *p* = 0.006, *η_p_^2^* = 0.188]. These data are shown in Figure 6 (panel B). Finally, the same analysis was conducted on the ‘evaluation reactivity index,’ which incorporates both commission and PES into a normalized score. There was no main effect of level of test anxiety [F(1, 37) = 0.27, *p* > 0.05, *η_p_^2^* = 0.007] or feedback [F(1, 37) = 0.01, *p* > 0.05, *η_p_^2^* = 0.0003]. Critically, there was a strongly significant interaction between these terms [F(1, 37) = 16.45, *p* = 0.0002, *η_p_^2^* = 0.308]. These data are shown in Figure 6 (panel C). Following these analyses, we conducted additional analyses to ensure that these findings did not reflect generalized performance deficits rather than evaluation-specific executive control disruption. To address this possibility, we examined overall go accuracy and no-go accuracy as dependent variables using the same 2-way ANOVA approach. The go accuracy, as expected, showed no main effect of level of test anxiety [F(1, 37) = 0.196, *p* > 0.05, *η_p_^2^* = 0.005], although there was a significant main effect of feedback [F(1, 37) = 12.06, *p* = 0.001, *η_p_^2^* = 0.246]. Post-hoc analyses showed that negative feedback increased go accuracy independent of level of test anxiety. Importantly, there was not a significant interaction between factors [F(1, 37) = 1.21, *p* > 0.05, *η_p_^2^* = 0.032]. Similarly, on no-go accuracy, there was no main effect of level of test anxiety [F(1, 37) = 0.066, *p* > 0.05, *η_p_^2^* = 0.002] or feedback [F(1, 37) = 1.42, *p* > 0.05, *η_p_^2^* = 0.037]. Again, importantly, there was not a significant interaction between factors [F(1, 37) = 0.368, *p* > 0.05, *η_p_^2^* = 0.01].

### 3.5 Discrimination from ADHD

**Figure 7:**
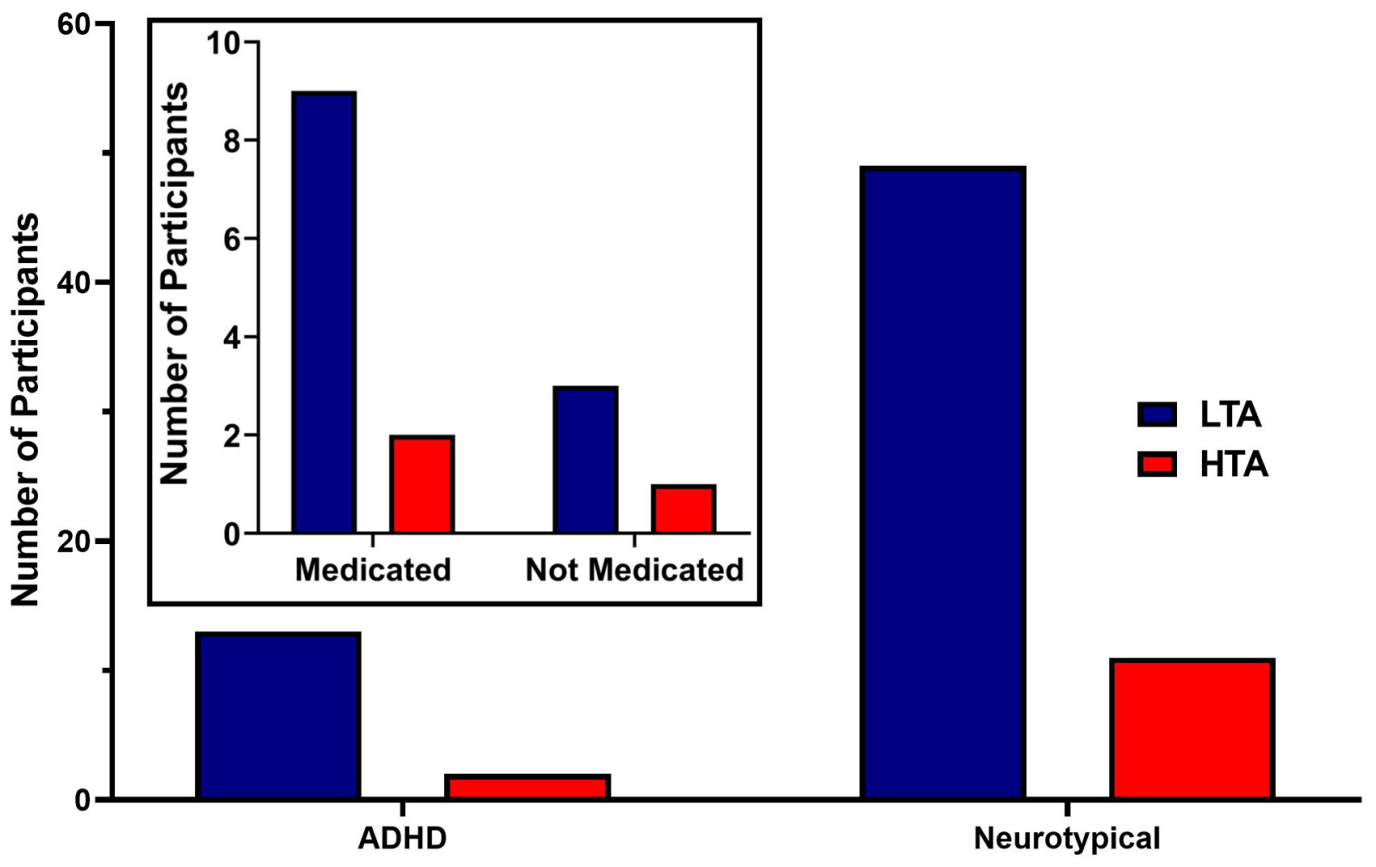
Number of participants classified by the PEAT as HTA or LTA and whether they had a previous diagnosis of ADHD (or ADD). Inset shows the number of participants with a diagnosis of ADHD classified by the PEAT as HTA or LTA, whether they were currently taking medication for the ADHD. There were no significant differences in the proportion of participants with or without ADHD (and those medicated or not) based on the results of the PEAT.

Seventy-five participants answered questions pertaining to previous diagnoses of either ADD or ADHD (from now on referred to as ADHD) as well as current medication use for ADHD (regardless of ADHD diagnosis). Of those surveyed, 15 (20%) reported a previous diagnosis of ADHD and of those, 11 were currently taking medication. None of those without a diagnosis of ADHD reported current use of ADHD medications. All participants currently medicated reporting taking either amphetamine, methylphenidate, or one of their derivatives (e.g., lisdexamfetamine). Among those classified as LTA (62 participants), 13 were diagnosed with ADHD whereas among those classified as HTA (13 participants), 2 were diagnosed with ADHD. Fisher’s exact test was performed to determine if there was an association between level of test anxiety classification based on the PEAT and a previous diagnosis of ADHD. The *p*-value from Fisher’s exact test was > 0.99 (*φ* = 0.053), suggesting that there was no association between a previous diagnosis of ADHD and being classified as HTA on the PEAT. To determine if medications typically used to treat ADHD reduced (or increased) the likelihood of being classified as HTA on the PEAT, another Fisher’s exact test was calculated among the participants with a previous diagnosis of ADHD based on whether they were currently medicated or not. Again, the p-value was > 0.99 (φ = 0.059), suggesting that there was no association between currently taking ADHD medication and being classified as HTA or LTA on the PEAT. These data are shown in Figure 7.

## 4 Discussion

The goal of this work was to develop and validate a computerized task that quantifies TA using the theoretical framework of the attentional control theory of anxiety. According to the ACT, HTA individuals should have an attentional bias towards, or a decrease in attentional control from the threat of a negative performance evaluation, requiring the allocation of more cognitive resources to compensate. If additional cognitive resources are unavailable, a measurable decrease in task performance would be expected. By definition, anxiety is only considered a disorder if it significantly interferes with some important area of functioning (in this case, test performance). Therefore, the approach taken to develop the PEAT was to objectively measure performance impairment induced by test-specific threatening information (i.e., negative performance feedback) during an attentionally demanding task.

A primary goal of the cortisol study was to determine whether PEAT classification corresponded to differences in physiological stress reactivity during performance evaluation. Consistent with this prediction, participants classified as HTA exhibited significantly greater cortisol increases than LTA participants despite the brief nature of the task and the absence of any meaningful academic consequences. These findings suggest that the PEAT identifies individuals who differ not only in performance disruption during evaluation, but also in physiological responsiveness to evaluative threat. Importantly, the −15% threshold initially identified from the score distribution and expected prevalence estimates produced clear physiological differentiation between groups, supporting its utility as a classification threshold. Salivary cortisol reactivity therefore served as an external physiological criterion against which candidate thresholds could be evaluated. More broadly, these findings address the criticism that there is no evidence of objective physiological responses in test anxious individuals to the testing environment compared to non-anxious peers (LeBeau et al., 2010). Notably, cortisol levels in LTA participants remained unchanged following the PEAT.

A second approach to validate the PEAT was to determine whether LTA and HTA differed in their attentional responses to test-specific threat (TST) relative to general threat (GT). Behaviorally, LTA demonstrated positive attentional engagement scores along with negative attentional disengagement scores to both GT and TST, indicating rapid orienting toward threat followed by efficient disengagement. LTA disengaged more effectively from GT than TST, resulting in a relative bias toward general threat and suggesting flexible, context-dependent attentional allocation. In contrast, HTA exhibited flattened engagement and disengagement scores, suggesting reduced flexibility in attentional control. The EEG data further clarify these behavioral findings by dissociating early attentional selection from later control-related processes. Both groups exhibited larger N2pc amplitudes to TST than GT items, indicating that test-specific threat preferentially captures attention at early stages, likely due to its personal relevance in a student sample. Although both groups exhibited preferential early attentional capture by TST, later control-related processes diverged substantially between LTA and HTA. Consistent with predictions from the ACT, HTA showed greater FMθ and TBR responses to TST than GT, whereas the opposite pattern was observed in LTA. FMθ has been associated with the recruitment of control-related resources, while elevated TBR has been linked to reduced attentional control and increased cognitive demands (Cavanagh and Frank, 2014; Putman et al., 2014; Morillas-Romero et al., 2015; Angelidis et al., 2018; Angelidis et al., 2016). Together, these findings suggest that HTA recruited greater control-related resources when confronted with evaluation-relevant threat, consistent with observations in other forms of anxiety (Cavanagh et al., 2017; Cavanagh and Shackman, 2015; Osinsky et al., 2017; Schmidt et al., 2018; McLoughlin et al., 2022). HTA exhibited greater Pθ responses to TST than GT, consistent with enhanced processing of evaluation-relevant threat, whereas LTA showed reduced Pθ and elevated Pα responses to TST. Given that Pθ has been associated with threat evaluation and Pα with the suppression of task-irrelevant information (Zhong et al., 2023; Aftanas et al., 2002; Knyazev et al., 2009; Arana et al., 2022; Bacigalupo and Luck, 2022; Jensen and Mazaheri, 2010), these findings suggest that LTA were more effective at suppressing the processing of TST. Together, these findings suggest that HTA and LTA differ not only in behavioral responses to threat, but also in the neurophysiological mechanisms underlying the allocation of attentional control during threat processing.

A third approach to validating the PEAT involved determining whether PEAT classification predicted responses to negative performance feedback in an unrelated go/no-go task. Participants classified as HTA exhibited greater disruption of executive control following feedback, including increased commission errors (reflecting reduced inhibitory control) and decreased post-error slowing (reflecting reduced adaptive updating). In contrast, LTA maintained relatively stable inhibitory control and showed evidence of enhanced adaptive adjustment following errors. These effects were captured by the evaluation reactivity index (ERI), which integrated feedback-related changes in commission error rate and PES, revealing a robust interaction between PEAT classification and feedback condition. Importantly, the differences in commission error rate and PES occurred in the absence of group differences in go or no-go hit rates, indicating that PEAT classification was associated with differential responses to evaluation rather than baseline differences in task performance.

Taken together, the cortisol, dot-probe, EEG, and go/no-go findings provide convergent and predictive evidence supporting the validity of the PEAT across multiple independent domains. Importantly, neither the dot-probe task nor the go/no-go task resembled the PEAT itself, indicating that PEAT classification generalized beyond the specific demands of the original task. Individuals classified as HTA and LTA differed in physiological stress reactivity, attentional processing of evaluation-relevant information, neural indices of attentional control, and executive responses to negative performance feedback. This convergence across behavioral, endocrine, and neurophysiological measures provides evidence that PEAT classification reflects meaningful individual differences in responses to evaluative threat rather than task-specific performance characteristics.

Finally, because the PEAT was developed using the ACT as its theoretical underpinning, it was important to determine whether the task differentiated attentional disruption due to test anxiety rather than other conditions associated with attentional control deficits, particularly ADHD. If the PEAT primarily measured attentional control deficits regardless of their source, a substantially larger proportion of participants with ADHD would be expected to be classified as HTA. However, only 2 of those with ADHD (13%) were classified as HTA, similar to (and lower than) the proportion of those without ADHD classified as HTA (18%). One potential explanation for these results could be that participants adequately treated for ADHD may not have the typical attentional problems associated with ADHD; therefore, the loss of attentional control measured by the PEAT would not be seen. Although there were only four participants with ADHD that were not currently taking any medication, only one of them was classified as HTA. Although the number of participants with ADHD was relatively small, these findings provide preliminary evidence that PEAT classification is not strongly associated with ADHD diagnosis. Thus, the attentional disruption identified by the PEAT appears to reflect responses to evaluative threat rather than generalized attentional control deficits alone. Future studies with larger ADHD samples will be important for more definitively establishing discriminant validity.

## 5 Limitations and future directions

There are two important limitations of the PEAT based on the currently available data. First, the normative score distribution and cutoff score require additional validation. All participants included in the studies reported here were college-aged, and although the cutoff score was supported by physiological differences in cortisol reactivity and was consistent with expected prevalence estimates of test anxiety, it was derived from the same datasets used to develop the classification scheme. Future studies should examine the stability and generalizability of both the normative distribution and cutoff score in independent samples across different age groups and educational settings. Secondly, the task was designed using colored targets and distractors and in all of the studies reported here, participants were excluded if they reported being colorblind. Therefore, the utility of the PEAT is unknown in this population. Future work should determine if colorblindness significantly impacts the normative scoring on this task, or a modified version of the task that uses grayscale or Gabor patches could be developed and validated specifically for the colorblind.

As mentioned above, the number of participants in the ADHD discrimination study was relatively small (*n* = 75). Although 13% of the ADHD participants were classified as HTA (which was lower than the non-ADHD population) and the study was powered to detect a proportion difference of 0.20 between groups (i.e., 15% of the non-ADHD population with PEAT score ≤ − 15% vs. 35% of ADHD population with the same PEAT score), larger samples will be necessary to improve confidence that the PEAT does not detect the loss of attentional control due to the attentional deficits typical of ADHD.

Finally, although the present findings suggest that PEAT classification is associated with altered responses to test performance evaluation, the specificity of the construct measured by the PEAT remains to be established. Negative evaluation is also a central feature of social anxiety disorder and other forms of evaluation-related anxiety. The present data cannot determine whether the PEAT measures test anxiety specifically or a broader form of evaluation anxiety. Future studies should therefore directly compare responses to test-performance evaluation and interpersonal evaluation, as well as examine the relationship between PEAT performance and established measures of social anxiety, fear of negative evaluation, and test anxiety. Such studies will be important for clarifying the specificity of the PEAT and further establishing its convergent and discriminant validity.

In conclusion, the PEAT is a computerized, objective task designed to quantify performance disruption under conditions of negative performance evaluation. Across multiple independent studies, PEAT classification was associated with differences in physiological stress reactivity, attentional processing of evaluation-relevant information, neural indices of attentional control, and executive responses to negative performance feedback. These findings provide convergent evidence supporting the validity of the PEAT. Grounded in the attentional control theory of anxiety and cognitive neuroscience, the task is easy to administer, takes less than 5 min to complete, and is easy to score. The present findings provide initial evidence that the PEAT captures domain-specific processes consistent with test anxiety; however, further work is needed to establish its generalizability across populations and its convergent and discriminant validity relative to other anxiety-related constructs.

## Data Availability

The datasets presented in this study can be found in online repositories. The names of the repository/repositories and accession number(s) can be found at: https://doi.org/10.7910/DVN/NXDZMA.
